# Machine Learning Integrates Bulk and Single‐Nucleus RNA Sequence to Explore Apoptosis‐Related Gene in Myocardial Infarction

**DOI:** 10.1155/cdr/5553167

**Published:** 2026-03-18

**Authors:** Bin Li, Yuan Wu, Shude Liao, Jing Wang, Panting Wei, Chenghui Yan, Dali Zhang, Dan Liu, Yaling Han

**Affiliations:** ^1^ Department of Cardiology, Renmin Hospital of Wuhan University, Wuhan, China, rmhospital.com; ^2^ State Key Laboratory of Frigid Zone Cardiovascular Diseases, Cardiovascular Research Institute and Department of Cardiology, General Hospital of Northern Theater Command, Shenyang, China, syjqzyy.com

**Keywords:** bioinformatics, machine learning, myocardial infarction, TNFRSF12A

## Abstract

**Background:**

Myocardial infarction (MI) remains a predominant contributor to global cardiovascular disease death, with apoptosis playing a pivotal yet incompletely characterized role in adverse cardiac remodeling. Despite advances in bulk RNA sequence analyses, the single‐cell level analyses of apoptotic signaling of MI progression remain unclear.

**Methods:**

Multiomics data integration was synthesized through single‐nucleus RNA‐seq (snRNA‐seq) (GSE270788) and bulk RNA‐seq (GSE21610). Three hundred ninety‐seven ARGs (apoptosis‐related genes) were downloaded from GeneCards (relevance score > 7), followed by identification of candidate genes through AUCell scoring and correlation analysis. Random Forest, Boruta, and LASSO algorithms were employed to refine hub genes. Hub genes were validated by animal and cell experiments.

**Results:**

Single‐nucleus sequence analysis reveals dramatic variation in ARGs activity across cardiac cells after MI, with fibroblasts demonstrating significantly elevated apoptotic activity compared with other cell types. Integration of single‐nucleus and bulk sequence confirms TNFRSF12A as the major molecule regulating ARGs activation in MI. Experiment validation confirmed that upregulation of TNFRSF12A in the MI model and TGF‐*β* induced NIH3T3.

**Conclusion:**

This study revealed that fibroblast dominates apoptotic activity post‐MI. We identified TNFRSF12A as a key regulator in MI through multiomics analysis, with potential as a diagnostic biomarker and therapeutic target.

## 1. Introduction

Myocardial infarction (MI) is a continuous process in which coronary artery obstruction leads to insufficient blood supply and myocardial ischemia, subsequently causing hypoxia and energy metabolism disorder in cardiomyocytes, resulting in decreased adenosine triphosphate (ATP) levels [1, 2]. Under conditions of ATP depletion, the dysfunction of the sodium–calcium exchanger within the cell leads to continuous accumulation of calcium ions, activating intracellular enzyme systems and ultimately leading to calcium overload and apoptosis [3].

Although cardiomyocytes represent approximately 30% of cardiac cellular content [4], the human heart predominantly comprises nonmyocyte populations. Notably, quantitative analyses reveal an eightfold to ninefold higher apoptotic incidence in noncardiomyocytes compared with their cardiomyocyte counterparts [5]. However, previous studies on cardiac apoptosis in the heart have almost entirely overlooked the role of noncardiomyocytes apoptosis in MI [6, 7]. Apoptosis of cardiomyocytes predominates as the cause of cardiomyocyte loss and accelerates cardiac remodeling and heart failure [8]. Noncardiomyocytes apoptosis also might deteriorate cardiac function in advanced stages, preserving nonmyocytes, especially macrophages and granulocytes, could ameliorate heart failure [9–11].

To systematically characterize apoptosis‐related gene (ARG) networks in MI, we developed an integrative multiomics framework combining single‐nucleus RNA sequencing (snRNA‐seq) with bulk transcriptomic profiling. By combining advanced machine learning algorithms with bioinformatics analyses, we identified potential key genes involved in apoptosis‐mediated myocardial injury. Furthermore, our computational findings were validated through experiments, providing robust evidence for the functional significance of this gene.

This study provides the first comprehensive characterization of ARGs activity heterogeneity at the single‐cell level in MI, revealing tumor necrosis factor (TNF) receptor superfamily member‐12A (TNFRSF12A) as a critical regulator in MI. Our study strengthens the role of noncardiomyocytes apoptosis after MI and emphasizes potential diagnostic biomarkers and therapeutic functions. These findings might develop targeted therapies for mitigating adverse cardiac remodeling of MI patients.

## 2. Methods

### 2.1. Data Acquisition

Single‐nucleus sequence (GSE270788) and bulk RNA sequence (GSE21610) datasets of MI were acquired from the Gene Expression Omnibus *(* https://www.ncbi.nlm.nih.gov/, accessed on November 4, 2024). Three hundred ninety‐seven ARGs using a stringent relevance score cutoff (≥ 7) were obtained from GeneCards website (https://www.genecards.org/, accessed on November 4, 2024).

### 2.2. SnRNA‐Seq Dataset Processing

The snRNA‐seq dataset (GSE270788) comprised cardiac samples from seven healthy donors and five MI patients (acute phase, < 3 months postevent). Quality control, normalization, and clustering were performed using Seurat (v4.3.0), followed by UMAP‐based dimensionality reduction. [12]. The QC criteria were as follows: 500 < nFeature_RNA < 6000, 1000 < nCount_RNA < 25,000, mitochondrial read percentage < 15%, and hemoglobin read ratio < 5%. After QC, 12,352 cells were retained for downstream analysis.

### 2.3. Evaluation of ARGs Activity

ARGs activity was quantified using the AUCell algorithm [13]. Cells were segregated into ARG‐high and ARG‐low populations at the median activity threshold. Highly correlated ARGs were identified through correlation analysis. The FindMarkers‐derived DEGs from ARG activity groups were integrated with correlation data to prioritize candidates for further investigation.

### 2.4. Enrichment Analysis

Hub gene biological functions and pathway associations were systematically characterized using Gene Ontology (GO) enrichment and KEGG pathway analysis [14].

### 2.5. Bulk‐Seq Dataset Processing

Bulk RNA‐seq datasets from eight healthy donors and nine MI patients (GSE21610) were processed using the “limma*”* package (v3.56.0) to identify differentially expressed genes (DEGs) with statistical significance [15].

### 2.6. Machine Learning Algorithms

Hub ARGs were prioritized through comparative machine learning analysis using Boruta [16], Random Forest (RF) [17], and least absolute shrinkage and selection operator (LASSO) [18] feature selection methods. The Boruta algorithm assessed feature importance through iterative comparisons between original features and their randomly permuted shadow counterparts. The RF algorithm constructed 5000 decision trees, and feature importance was ranked by averaging their results, yielding 15 candidate genes. LASSO introduced regularization to compress certain regression coefficients to zero, enabling automatic feature selection and retaining genes with the highest contribution to the target variable. Hub ARGs were identified through consensus analysis of three independent algorithms, with intersecting candidates visualized via Venn diagram.

### 2.7. Immune Infiltration

The single‐sample gene set enrichment analysis (ssGSEA) algorithm (*GSVA* package) quantified the relative abundance of 28 immune cell types [19]. Immune cell modules were derived from metagene sets [20].

### 2.8. Receiver Operating Characteristic (ROC) Analysis

The diagnostic performance of candidate hub genes was quantitatively assessed using ROC curve analysis, with the area under the curve (AUC) serving as a metric for predictive accuracy [21].

### 2.9. Gene Set Enrichment Analysis (GSEA)

TNFRSF12A expression in MI patient RNA‐seq (GSE21610, *n* = 9) stratified samples into high/low groups at the median. *Limma*‐based analysis identified DEGs between these subgroups [15]. Similarly, cardiomyocytes from five MI patients in the snRNA‐seq dataset (GSE270788) were classified into two subtypes (TNFRSF12A^+^ fibroblasts and TNFRSF12A^−^ fibroblasts), and differential expression analysis was conducted.

KEGG and Reactome pathway analyses were conducted using the clusterProfiler (v4.0) and enrichplot (v1.12.0) R packages, with gene sets curated from the MSigDB database (release 7.0; KEGG: c2.cp.kegg.v7.0.entrez.gmt; Reactome: c2.cp.reactome.v7.0.entrez.gmt).

### 2.10. Cell Communication

The average expression levels and interaction strengths of ligands and receptors were calculated using the default CellChatDB [22].

### 2.11. Animal Model

C57BL/6J mice (male 8 weeks old; GemPharmatech) were maintained under specific pathogen‐free conditions. All the mice were assigned into two groups: sham group (*n* = 6) and MI group (*n* = 6). MI group mice underwent left anterior descending (LAD) coronary artery ligation, whereas sham operated controls without LAD occlusion [23]. The study endpoint was set at 28 days post‐MI.

All experimental procedures were conducted in strict accordance with: NIH guidelines for the care and use of laboratory animals and ARRIVE guidelines (https://arriveguidelines.org). The study was approved by the Institutional Animal Care and Use Committee of General Hospital of Northern Theater Command (Ethical Approval No. 2024‐84).

### 2.12. Cell Treatment

NIH3T3 fibroblasts (IM‐M050, Xiamen Immocell Biotechnology Co. Ltd.) were treated with human TGF‐beta 1 Recombinant Protein (TGF*β*, 10 ng/mL, 100‐21C‐50UG, PeproTech) for 24 h.

### 2.13. Echocardiography Assessment

For echocardiography, mice were pre‐anesthetized (2% isoflurane/0.5 L/min O_2_) 24 h prior to minimize stress artifacts. After depilation, steady‐state anesthesia was maintained (1%–1.5% isoflurane/0.5 L/min O_2_) with depth confirmed by absent pedal reflexes. Cardiac phenotyping at 28 d post‐MI used high‐resolution ultrasound (Vevo 2100, FUJIFILM) [24, 25]. B‐mode imaging captured anatomical morphology, whereas M‐mode tracked myocardial motion dynamics. Euthanasia was applied by cervical dislocation under deep anesthesia (3% isoflurane in 100% O_2_ at 1 L/min for > 5 min).

### 2.14. Western Blot

Myocardial tissue and NIH3T3 cell were lysed on ice. Proteins were resolved by 10% SDS‐PAGE and transferred to PVDF membranes (EMD Millipore). After blocking with 5% skim milk (1 h, RT), membranes were incubated with: Anti‐TNFRSF12A (1 *μ*g/ml, A5955, goat antirabbit, and Abclonal), Anti‐GAPDH (2 *μ*g/ml, goat antirabbit, A19056, and Abclonal), and HRP‐conjugated secondary antibody (0.1 *μ*g/ml, goat antirabbit, AS014, and Abclonal) was applied for 1 h at room temperature. Band intensity was quantified (ImageJ) and normalized to GAPDH.

### 2.15. Real‐Time Quantitative Polymerase Chain Reaction (qPCR)

Total RNA was purified from myocardial tissue specimens (G3013, Servicebio, and Trizol). Reverse transcription was performed with 1 *μ*g total RNA input (G3337‐50, Servicebio, and SweScript All‐in‐One RT SuperMix for qPCR). Gene expression was quantified by qPCR (BIO‐RAD CFX96) using the following primers: *Tnfrsf*12*a* (mouse): F—5 ^′^‐GTGTTGGGATTCGGCTTGGT‐3 ^′^, R—5 ^′^‐GTCCATGCACTTGTCGAGGTC‐3 ^′^; *18S* (mouse, internal control): F—5 ^′^‐CGCCGCTAGAGGTGAA ATTC‐3 ^′^, R—5 ^′^‐TTGGCAAATGCTTTCGCTC‐3 ^′^.

### 2.16. Statistical Analysis

Bioinformatics analyses (R v4.3.0) and experimental data (Prism 9) were implemented with normality testing (Shapiro–Wilk) and appropriate comparative analyses (*t*‐test/Mann–Whitney test/Wilcoxon test), with *p* < 0.05 considered statistically significant. Analyze the correlation between variables using the Pearson correlation coefficient. Continuous data are expressed as mean ± SEM or median (25% percentile–75% percentile). Detailed statistical analysis can be found in Table S1.

## 3. Results

### 3.1. Analyzing snRNA‐Seq

**Figure 1:**
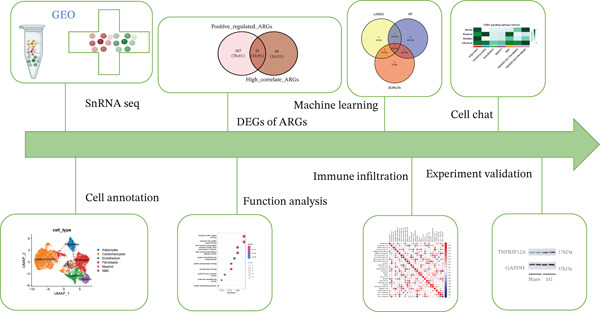
Workflow diagram.

The experimental workflow was schematically outlined in Figure 1. Twelve samples (seven donor and five patients with MI) from snRNA‐seq were assigned to analysis. Pre‐ and post‐QC comparisons of the 12 samples included: gene expression distribution, hemoglobin gene content (%), and mitochondrial gene fraction (%) (Figure 2a,b), indicating that QC filtered low‐quality cells. Batch effect correction analysis showed that global cell distribution was stable (Figure 2c). The cells were divided into 18 cell clusters, which were identified as six cell types (myeloid, smooth muscle cells (SMCs), cardiomyocytes, fibroblasts, endothelium, and adipocytes) after annotation of characteristic marker genes (Figure 2d, 2e, and 2f).

Figure 2Processing of single‐cell data. (**a)** Pre‐QC metrics for 12 samples: gene expression distribution, hemoglobin gene content, and mitochondrial gene abundance. (**b)** Post‐QC profiles showing improved data quality. (**c)** Batch effect correction efficiency (UMAP). (d–f) Unsupervised clustering (18 clusters) annotated to six cell types using canonical markers.

**Figure:**
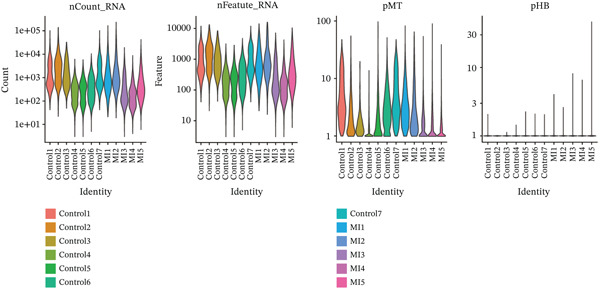
(a)

**Figure:**
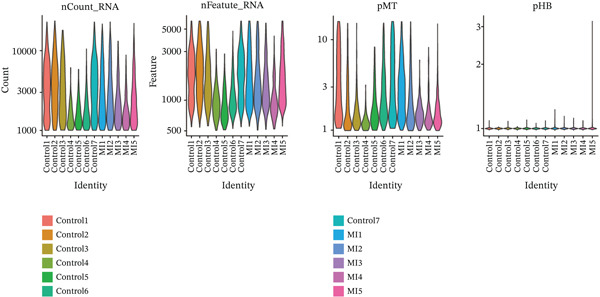
(b)

**Figure:**
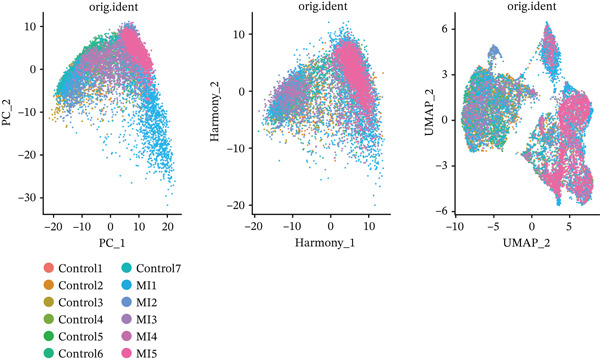
(c)

**Figure:**
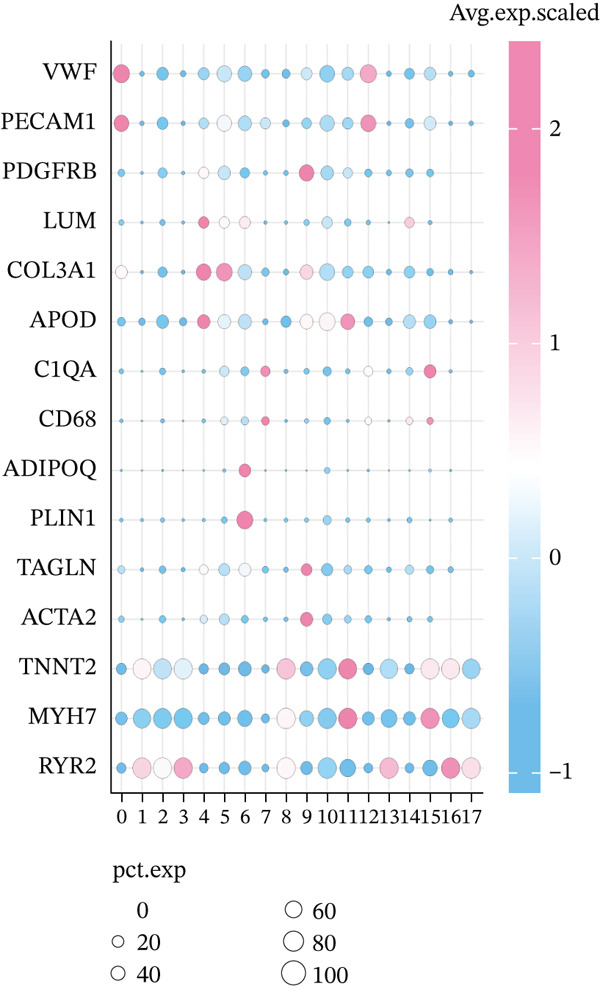
(d)

**Figure:**
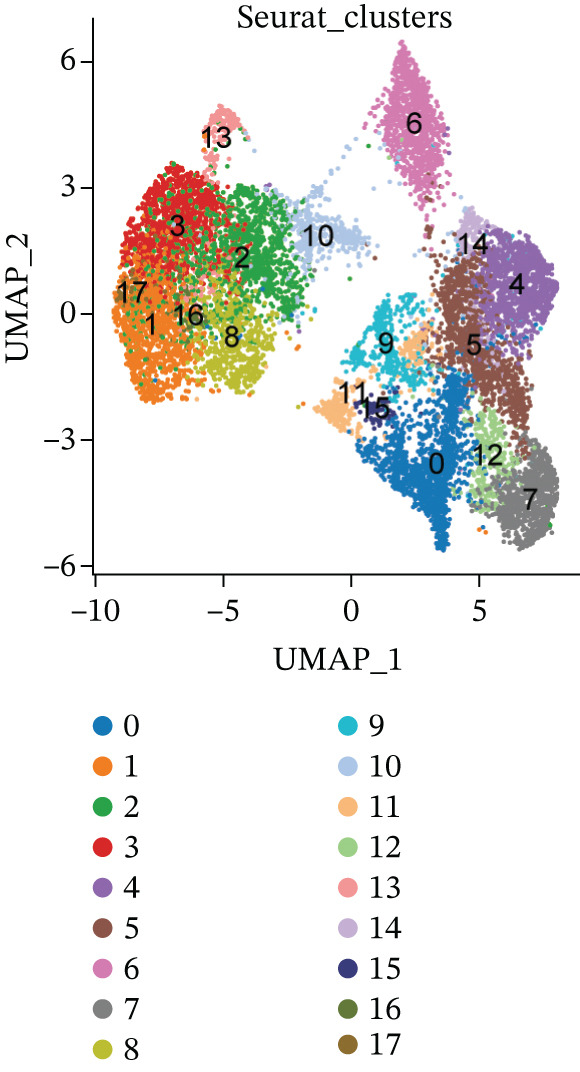
(e)

**Figure:**
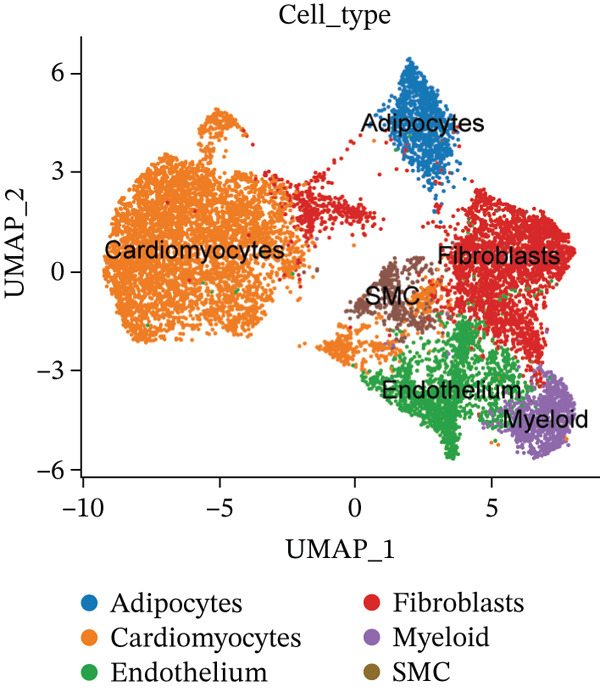
(f)

The cell proportion of two groups (control group and MI group) and every sample was observed (Figure 3a, 3b, and 3c). The numbers of myeloid, fibroblasts, endothelium, and adipocytes were increased in the MI group, whereas those of SMCs and cardiomyocytes were decreased in the MI group. The six cell populations were characterized by gene expression distributions, hemoglobin gene content (%), and mitochondrial gene abundance (%) (Figure 3d).

Figure 3Comparison of single‐cell data in two groups. (a–b) The cell proportion of two groups. (c) Contribution of 12 samples in six cell types. (d) Quality control metrics for single‐cell data.

**Figure:**
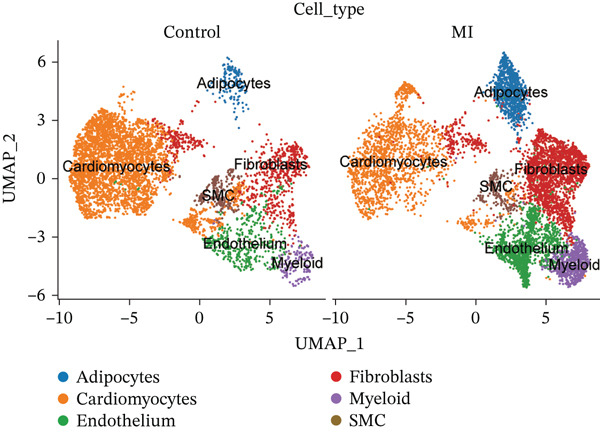
(a)

**Figure:**
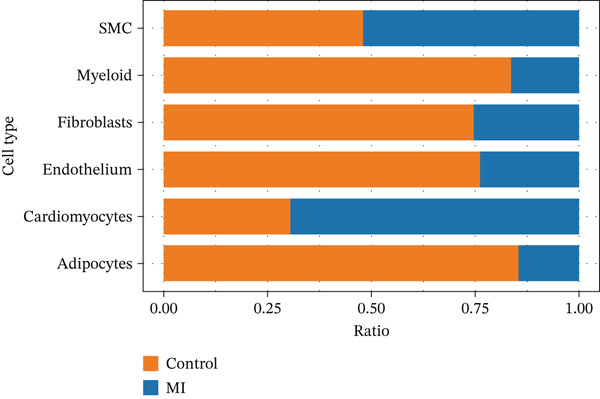
(b)

**Figure:**
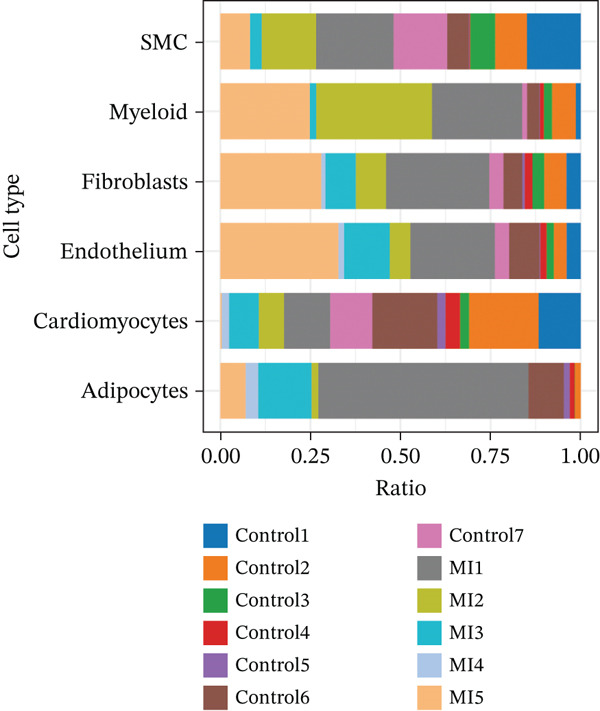
(c)

**Figure:**
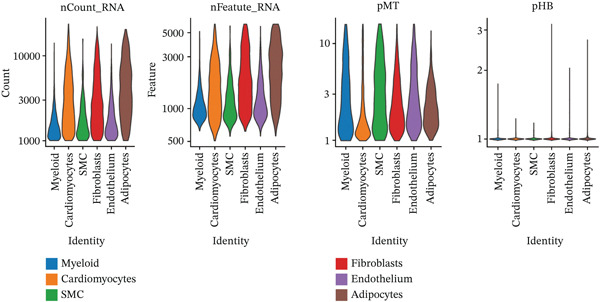
(d)

### 3.2. ARGs Activity Analysis

ARGs activity in MI group at single cell level was investigated. ARGs activity in single cell level was calculated by AUCell algorithm (Figure 4a, 4b, and 4c). The results demonstrated that all cells showed higher level ARGs activity in MI group than control group. In MI group, ARGs activity of fibroblasts was higher than other cell types except for adipocytes. Identification of top 100 genes exhibiting significant associations with ARG activity through correlation analysis.

Figure 4Heterogeneity among the expression of ARGs in cell level of MI group. (a–b) UMAP plot and violin plot of ARGs activity of two groups by AUCell algorithm. (c) Violin plot of ARGs activity in all cell types from control and MI group (left) and the ARGs activity score for fibroblasts compared with other cell types (right). (d–e) Filtration of Top 100 genes by correlation analysis. (f) Spatial mapping of ARG activity scores (high: red; low: blue) at single‐cell resolution. (g) Results of DEGs analysis of ARGs. (h) Venn plots of overlap between positively‐regulated ARGs and high‐correlated ARGs.

**Figure:**
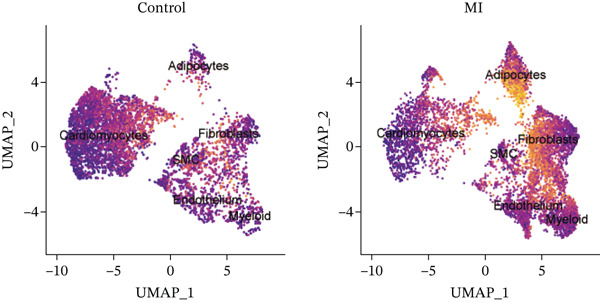
(a)

**Figure:**
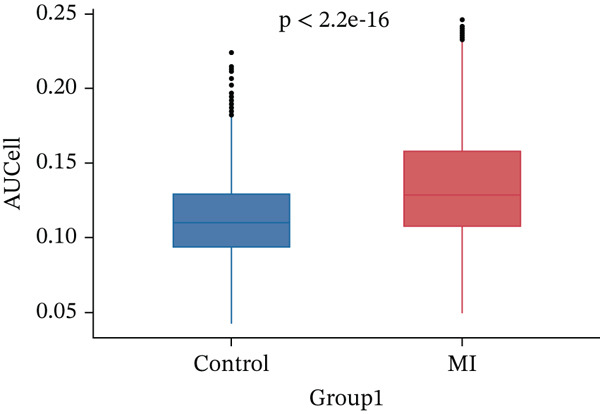
(b)

**Figure:**
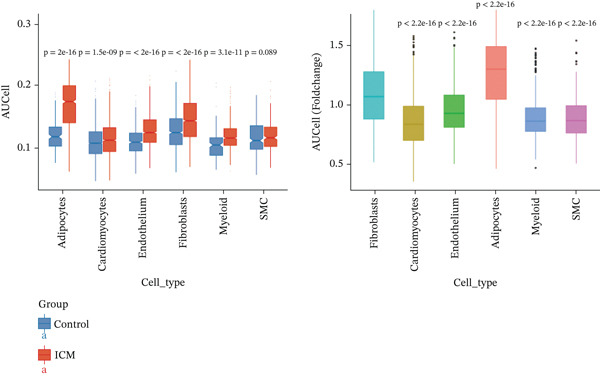
(c)

**Figure:**
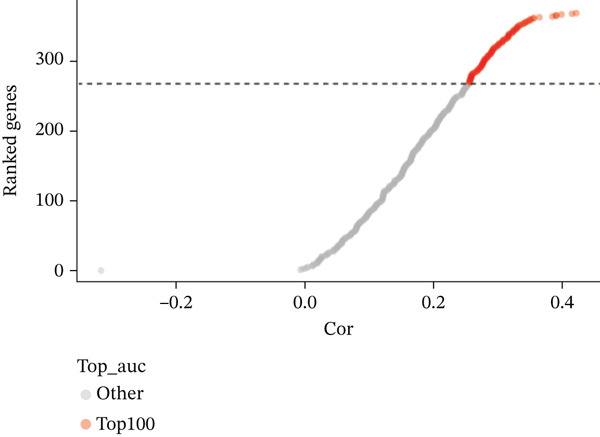
(d)

**Figure:**
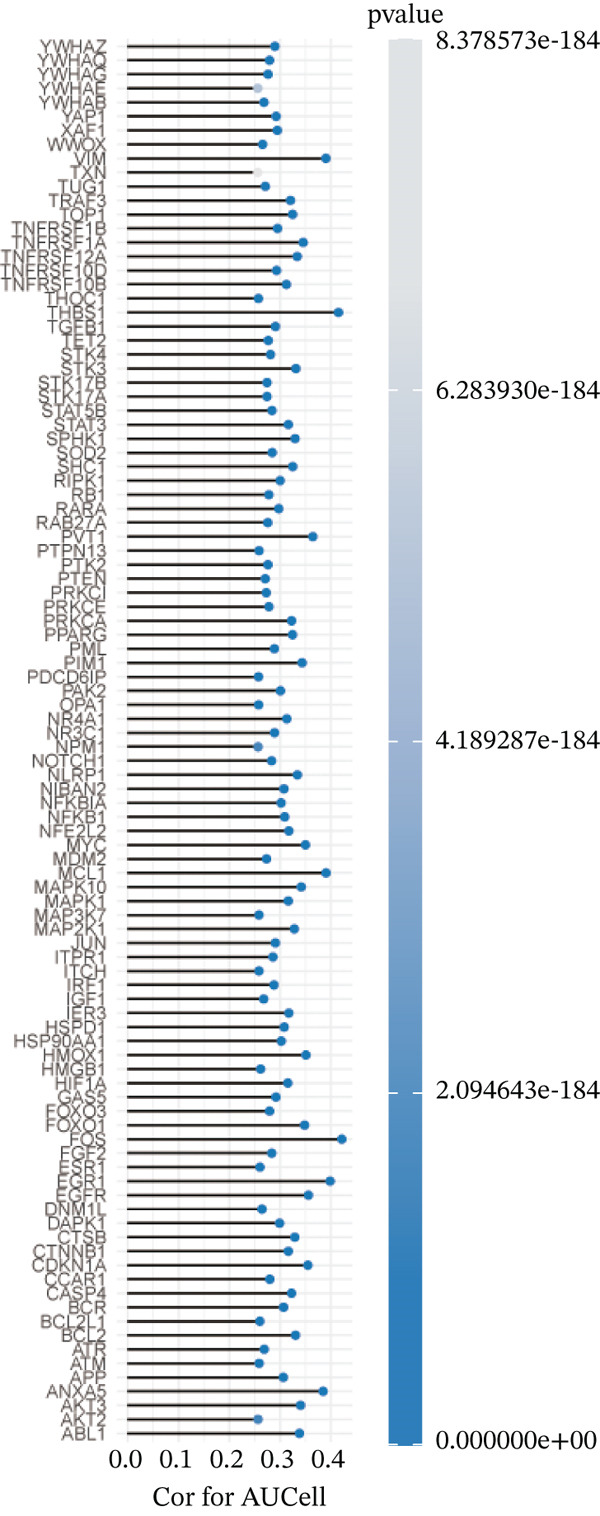
(e)

**Figure:**
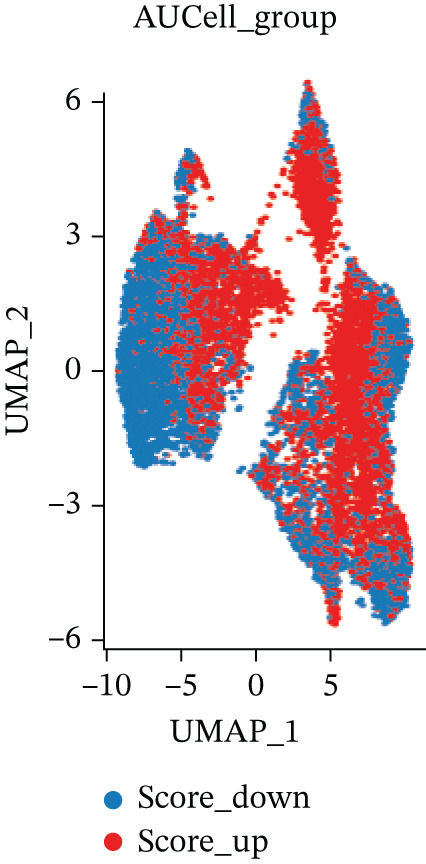
(f)

**Figure:**
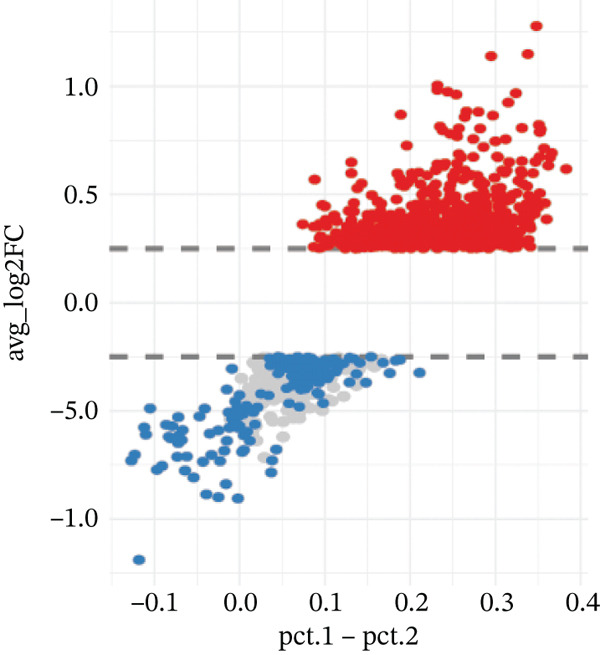
(g)

**Figure:**
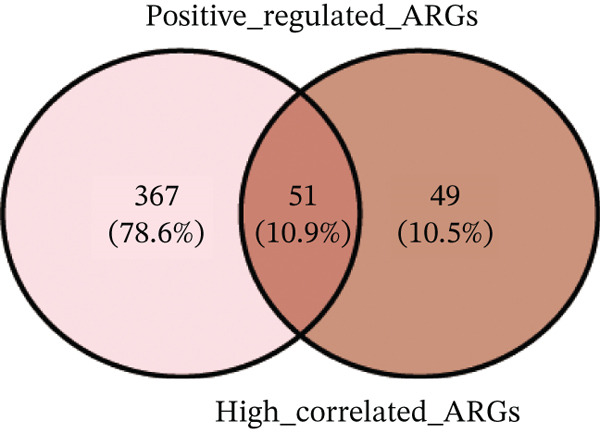
(h)

Correlation analysis was conducted to filter 100 top genes significantly related with ARGs activity (Figure 4d,e). Cells were stratified into high‐ and low‐activity groups based on median ARG expression scores. Four hundred eighteen upregulated DEGs were identified between the two groups (Figure 4f,g). Further, 51 genes overlapped between the Top 100 correlation analysis genes and 418 upregulated DEGs (Figure 4h).

### 3.3. KEGG and GO Enrichment Analysis

Fifty‐one genes were conducted for further GO and KEGG enrichment analysis. GO analysis identified significant enrichment in: biological process (BP)—regulation of apoptotic signaling; cellular component (CC)—RNA polymerase II transcription regulator complex; and molecular function (MF)—ubiquitin protein ligase binding (Figure 5a, 5b, and 5c). KEGG pathway analysis of the 51‐gene set revealed predominant involvement of the PI3K‐Akt signaling pathway (Figure 5d).

Figure 5Functional enrichment analysis of ARGs. (a) Biological process (BP) enrichment. (b) Cellular component (CC) enrichment. (c) Molecular function (MF) enrichment. (d) KEGG pathway enrichment analysis.

**Figure:**
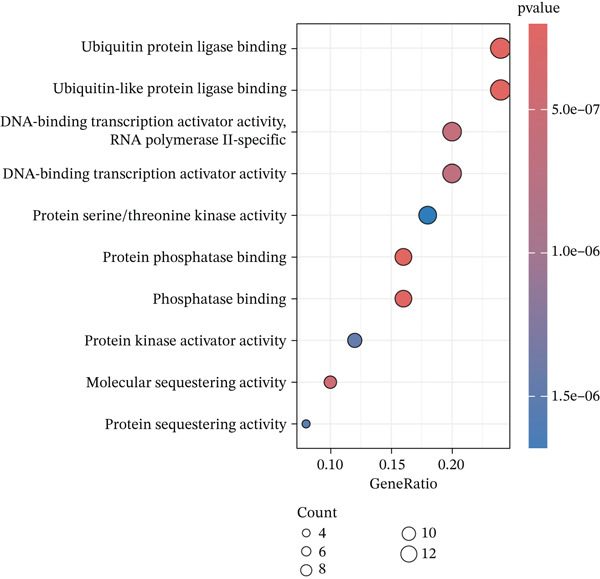
(a)

**Figure:**
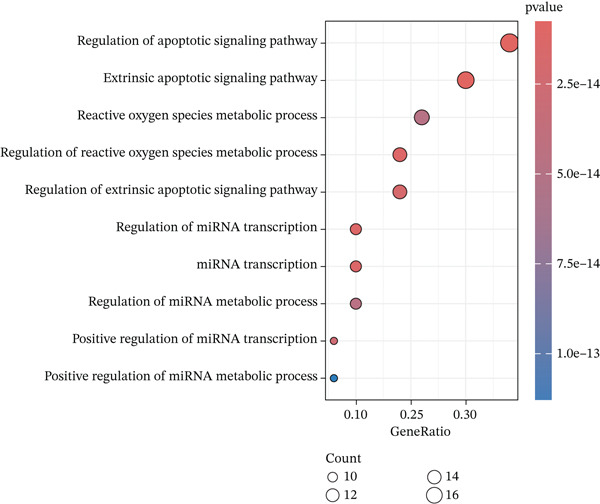
(b)

**Figure:**
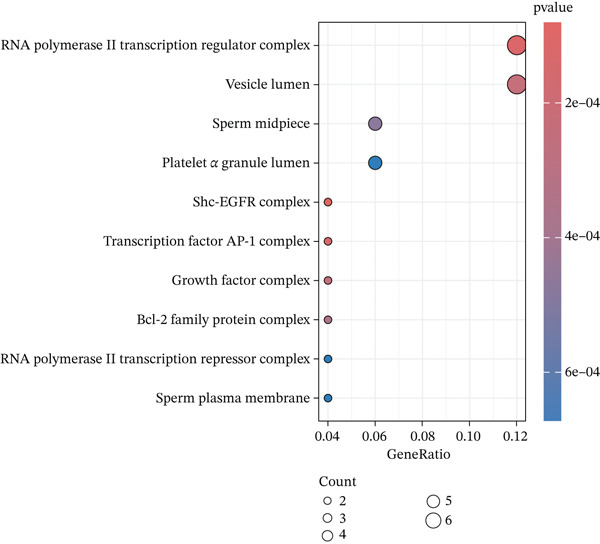
(c)

**Figure:**
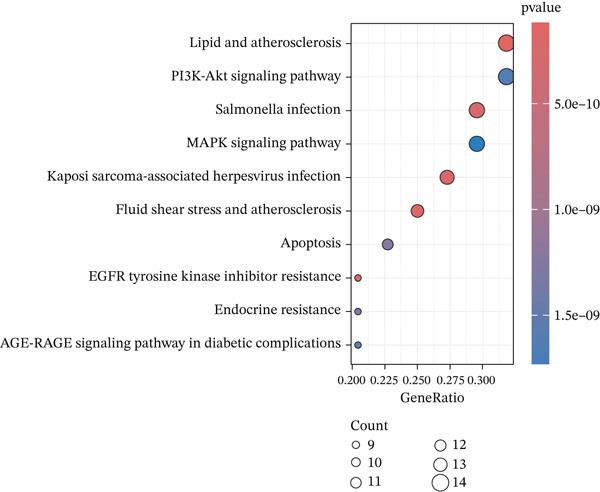
(d)

### 3.4. Identification of the Optimal Genes by Machine Learning

Raw data of eight donors and nine MI patients in GSE21610 was normalized (Figure 6a). Three distinct machine‐learning approaches—LASSO regression, Boruta feature selection, and RF analysis—were systematically employed to identify optimal gene signatures. Two hub genes were identified by LASSO algorithm (Figure 6b,c). Top 10 genes were identified after construction of 5000 decision trees by RF algorithm (Figure 6d). Three key genes were marked by Boruta algorithm (Figure 6e,f). Additionally, one optimal gene was filtered by merging results of three machine learning (Figure 6g).

Figure 6Machine learning‐based feature gene selection. (a) Box plots of Normalized raw data across 17 biological samples.(b–c) LASSO regression analysis. (d) Top 20 genes by RF classification. (e–f) Results of boruta algorithm. (g) Consensus genes identified through intersection of LASSO, RF, and Boruta outputs.

**Figure:**
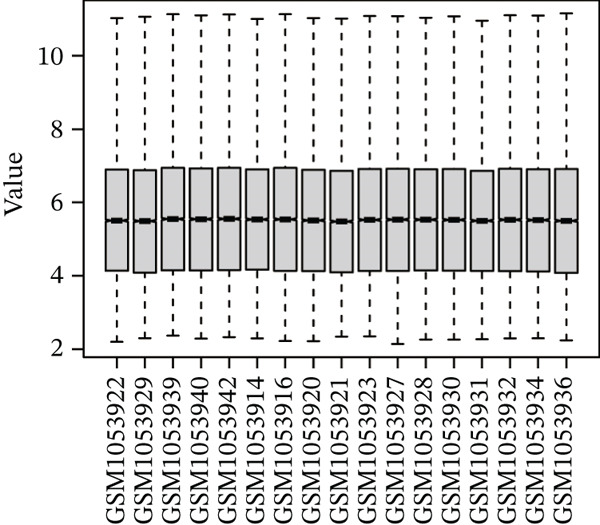
(a)

**Figure:**
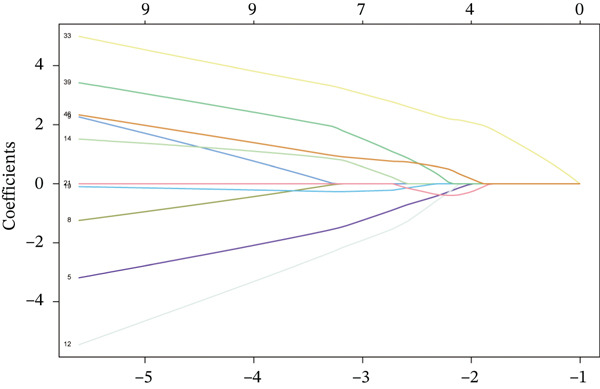
(b)

**Figure:**
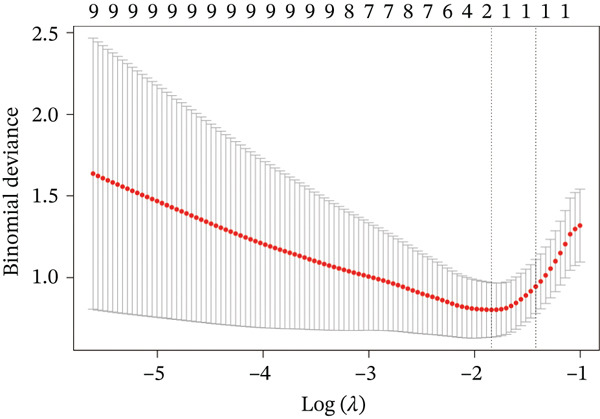
(c)

**Figure:**
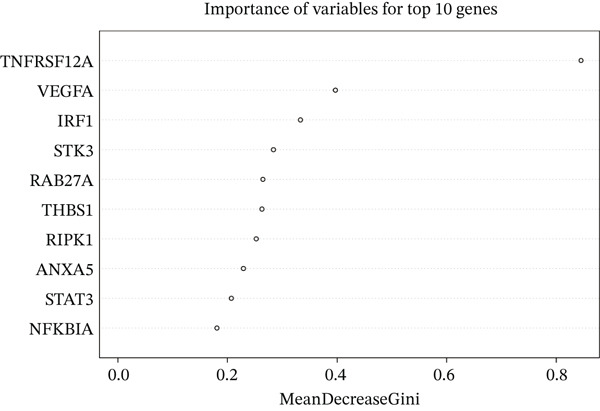
(d)

**Figure:**
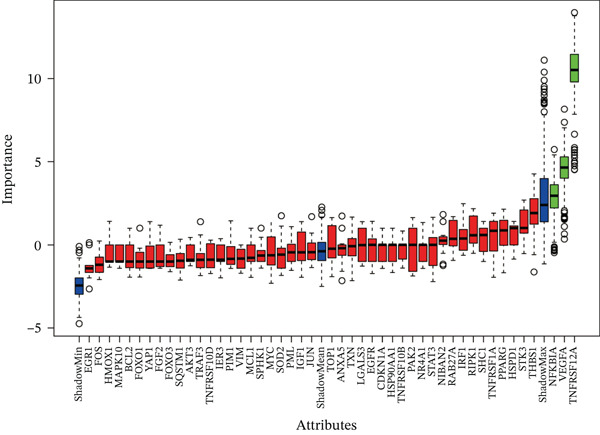
(e)

**Figure:**
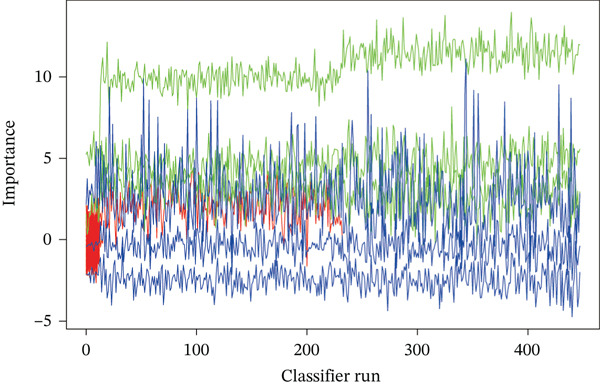
(f)

**Figure:**
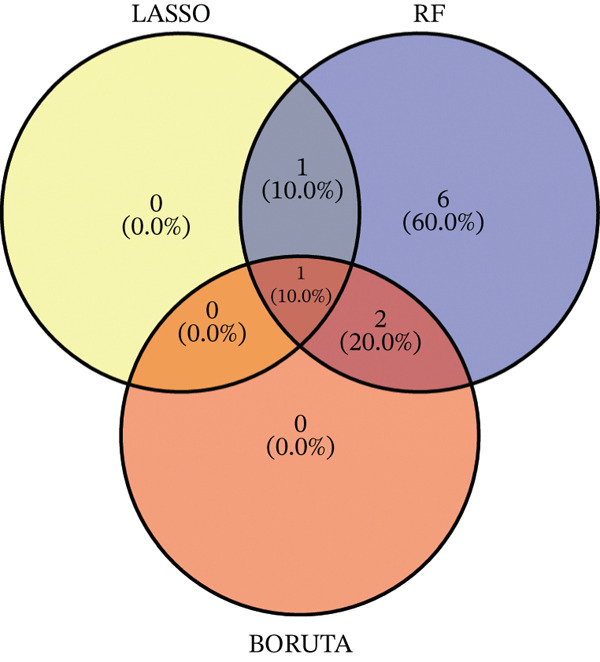
(g)

### 3.5. Immune Infiltration

Correlation matrix represented relation among the infiltrating immune cells (Figure 7a). TNFRSF12A gene was well correlated with a variety of immune cells and immune functions (Figure 7b). For instance, TNFRSF12A was positively correlated with immature dendritic cells and effector memory CD4^+^ cells. Transcript levels of TNFRSF12A in control and MI group (Figure 7c). Compared with control group, TNFRSF12A was upregulated in MI group. The AUC of TNFRSF12A was 0.983, which indicated substantial diagnostic value of TNFRSF12A for MI (Figure 7d).

Figure 7Immune landscape and functional enrichment of hub genes. (a) Correlation network of 28 immune cell types. (b) Hub gene‐immune cell interactions. (c) Expression level of hub genes between two groups. (d) ROC curve for TNFRSF12A. (e–h) UMAP plot, violin plot and ridge Plot of TNFRSF12A. (i–j) GSEA analysis of TNFRSF12A in Reactome and KEGG pathway.

**Figure:**
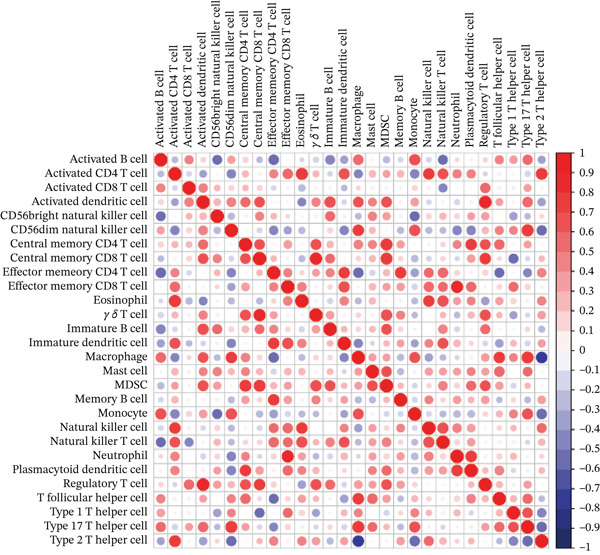
(a)

**Figure:**
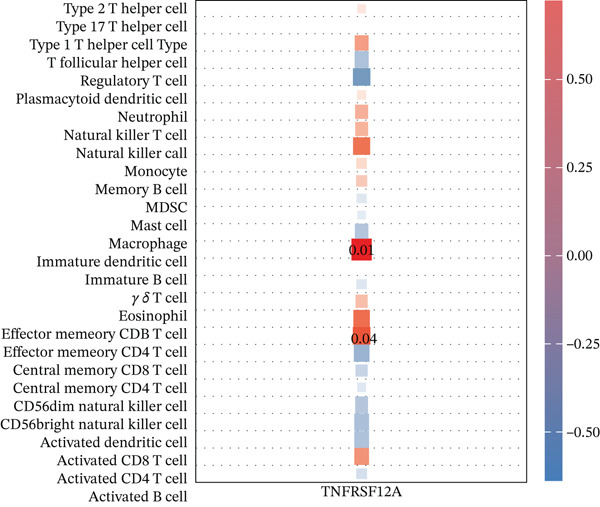
(b)

**Figure:**
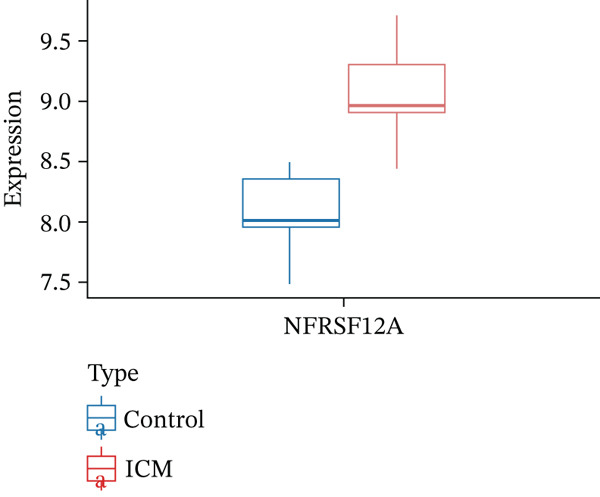
(c)

**Figure:**
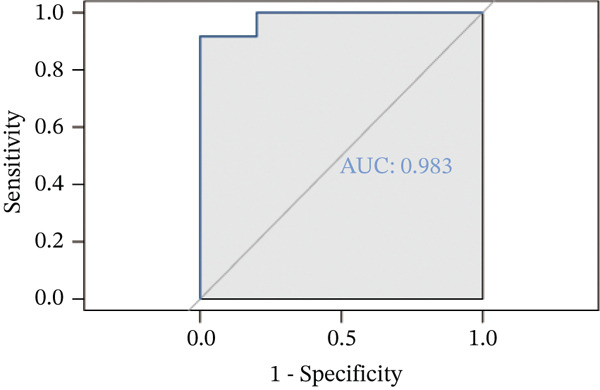
(d)

**Figure:**
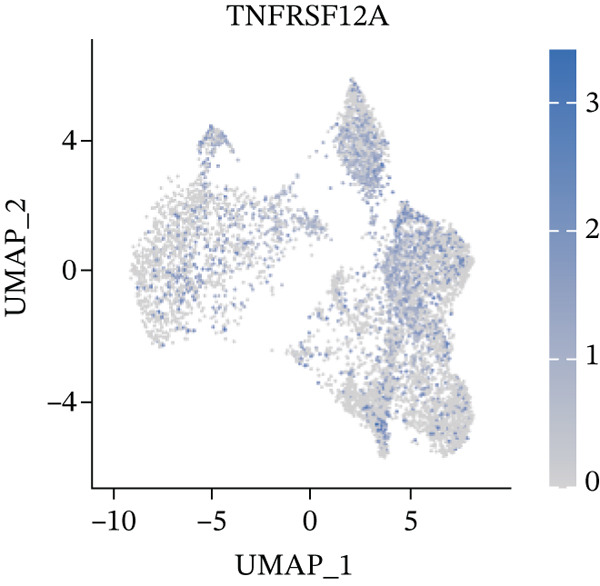
(e)

**Figure:**
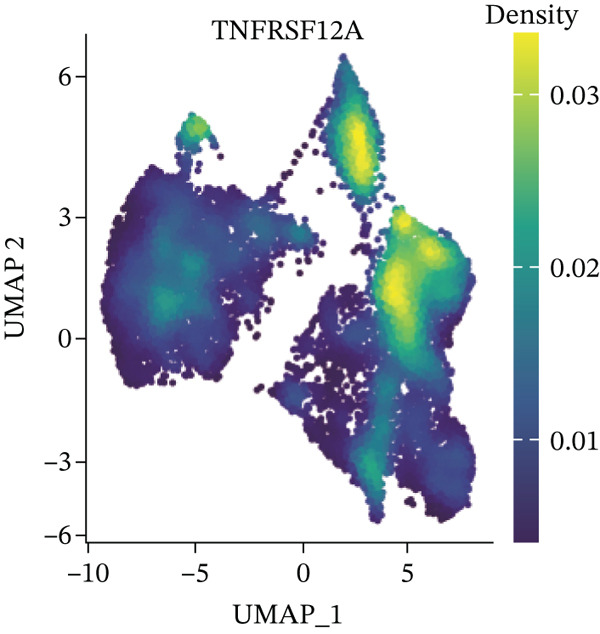
(f)

**Figure:**
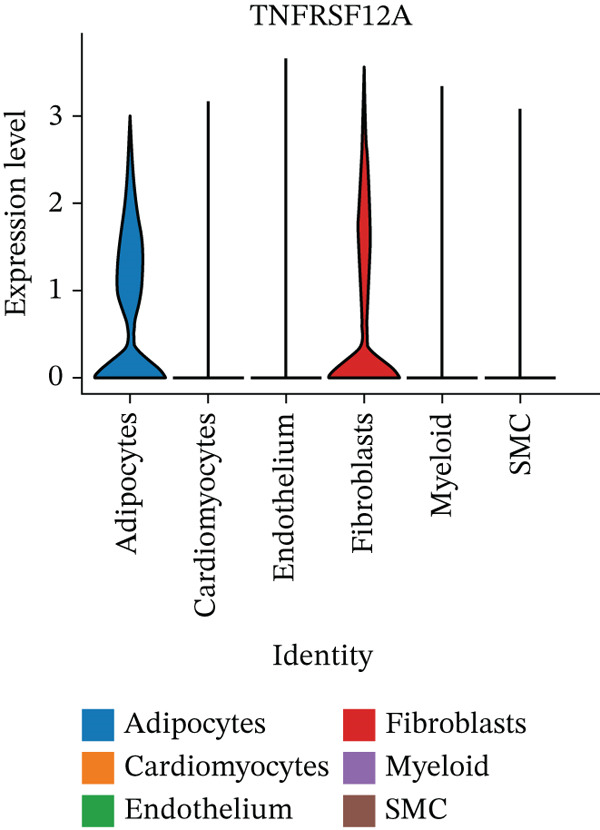
(g)

**Figure:**
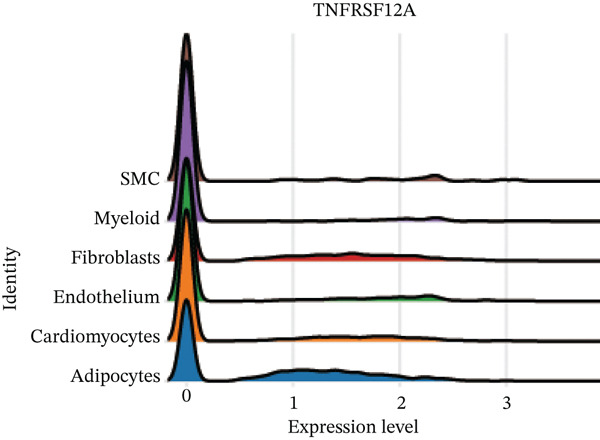
(h)

**Figure:**
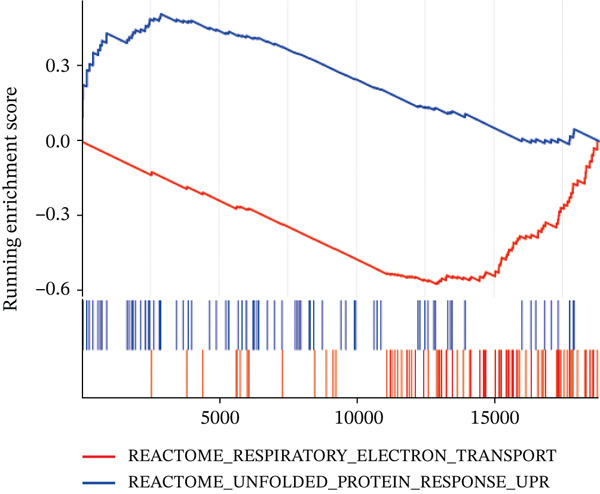
(i)

**Figure:**
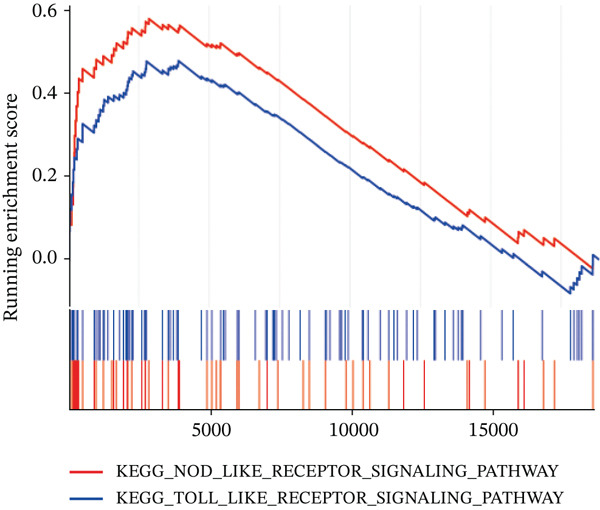
(j)

UMAP, ridge plot, and violin plot visualization showed that TNFRSF12A was primarily localized in the fibroblasts and the adipocytes (Figure 7e, 7f, 7g, and 7h). GSEA analysis of DEGs between TNFRSF12A high‐ and low‐expression groups revealed that TNFRSF12A played a role in nod‐like receptor and toll‐like receptor signaling, respiratory electron transport, and unfolded protein response (Figure 7i,j).

### 3.6. Cell Communication Analysis

Fibroblasts were classified into two distinct populations: TNFRSF12A^+^ and TNFRSF12A^−^ fibroblasts. Cell communication analysis revealed the number of interactions and their respective interaction weights between TNFRSF12A^+^ fibroblasts, TNFRSF12A^−^ fibroblasts, and other cell types (Figure 8a,b). Among the outgoing signaling pathways, CXCL, PROS, and SEMA4 were predominantly expressed in TNFRSF12A^+^ fibroblasts. In contrast, the incoming signaling pathways predominantly expressed in TNFRSF12A^+^ fibroblasts included CXCL, SEMA5, BMP, ADGRE5, and TENASCIN (Figure 8c). TNFRSF12A^+^ fibroblasts exhibited stronger incoming and outgoing signaling strengths compared with TNFRSF12A^−^ fibroblasts (Figure 8d,e). Ligand‐receptor interaction analysis between all cell types and TNFRSF12A^+^ or TNFRSF12A^−^ fibroblasts indicated that TNFRSF12A^+^ fibroblasts primarily communicated with other cells through the THBS1‐CD36 and THBS‐CD47 receptor‐ligand pairs (Figure 8f,g). Within the THBS signaling pathway, TNFRSF12A^+^ fibroblasts were identified as senders, whereas cardiomyocytes were identified as receivers (Figure 8h,i). Reactome analysis of DEGs between TNFRSF12A^+^ and TNFRSF12A^−^ fibroblasts suggested that TNFRSF12A might regulate syndecan interactions as well as interleukin‐4 and interleukin‐13 signaling pathways in fibroblasts (Figure 8j).

Figure 8Cell communication analysis of TNFRSF12A^+^ fibroblasts. (a) Circle plots shown that number of interactions and interaction weights among global cells. (b) Heat map of interaction weights among all cells. (c) Bidirectional signaling patterns (outgoing/incoming) for each cell cluster. (d) Cell‐type‐specific interaction dynamics. (e) Comparative analysis of outgoing signal capacity versus incoming signal reception in TNFRSF12A^+^ and TNFRSF12A^−^ fibroblasts. (f–g) Ligand‐receptor pairs (bubble plots) mediating crosstalk with TNFRSF12A^+^ and TNFRSF12A^−^ fibroblasts. (h) THBS‐specific intercellular communication networks. (i) THBS signaling pathway network. (j) GSEA analysis of TNFRSF12A^+^ fibroblasts.

**Figure:**
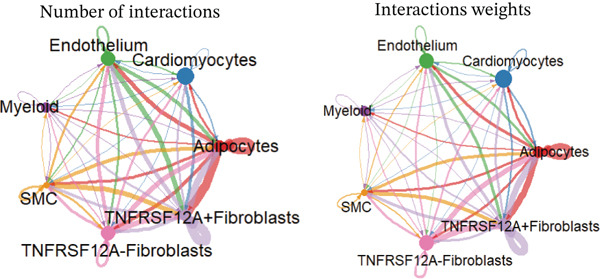
(a)

**Figure:**
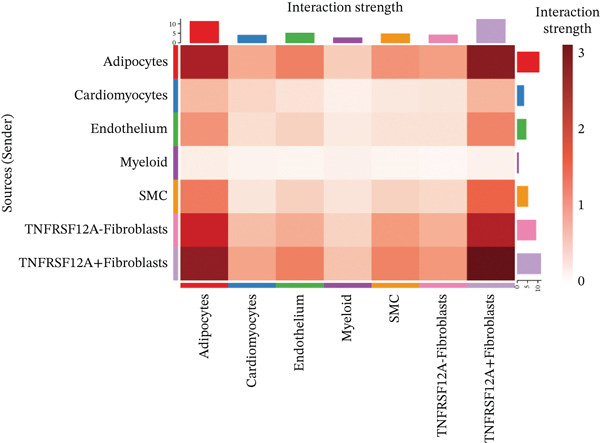
(b)

**Figure:**
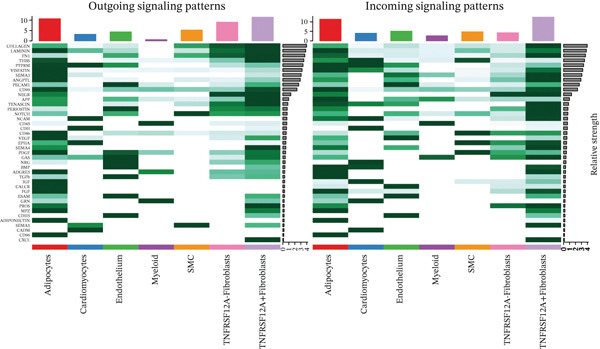
(c)

**Figure:**
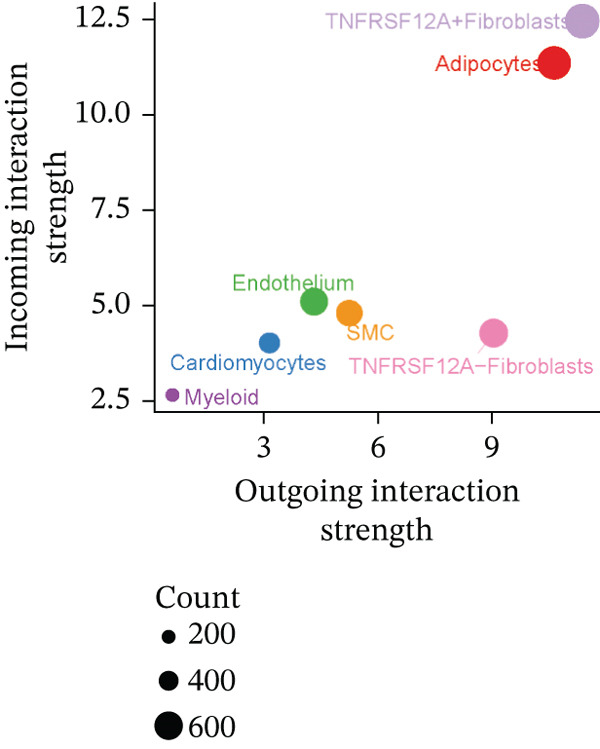
(d)

**Figure:**
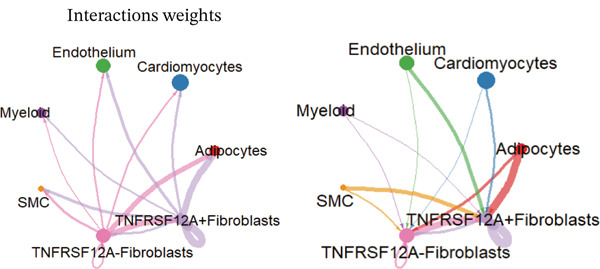
(e)

**Figure:**
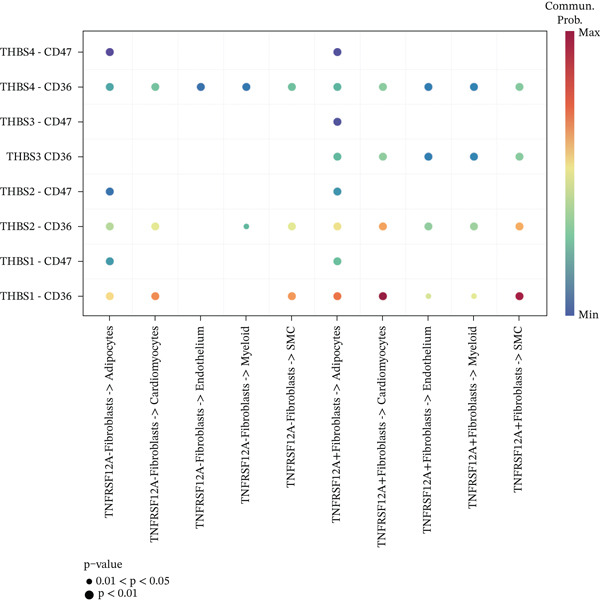
(f)

**Figure:**
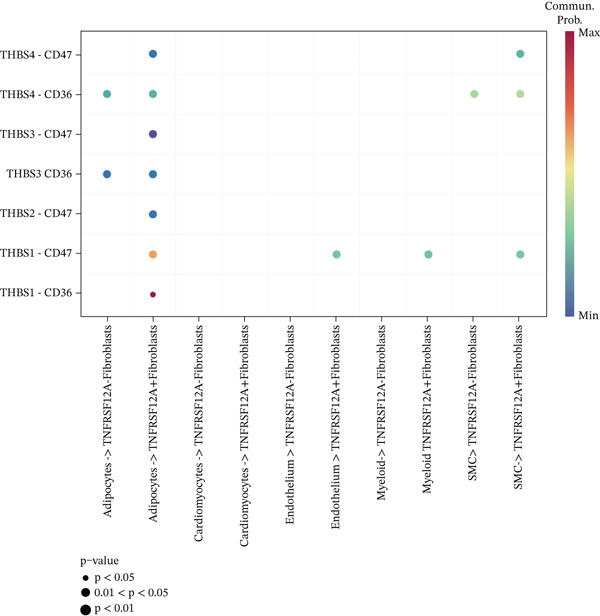
(g)

**Figure:**
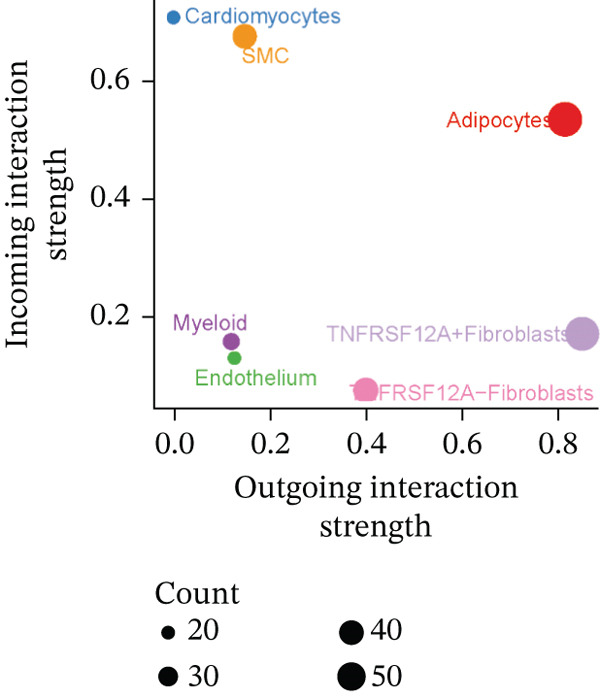
(h)

**Figure:**
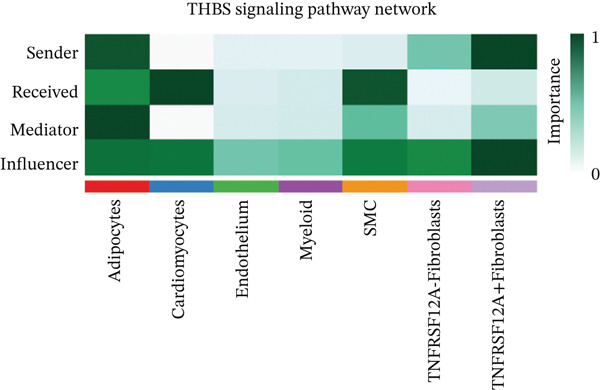
(i)

**Figure:**
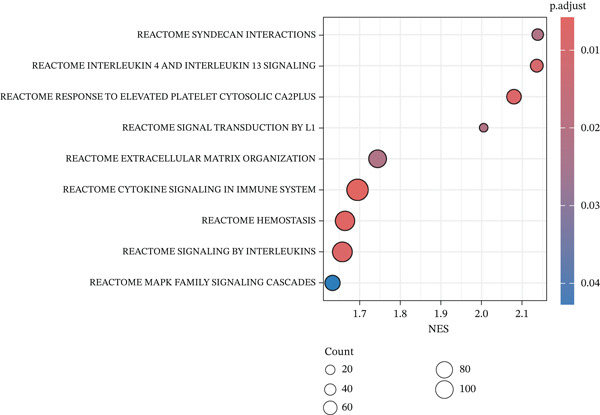
(j)

### 3.7. Experimental Validation of TNFRSF12A

Among fibroblasts, deletion of TNFRSF12A ameliorated pulmonary arterial hypertension induced right heart fibrosis and idiopathic pulmonary fibrosis [26, 27]. Engel et al. demonstrated that TNFRSF12A was a positive regulator of cardiac hypertrophy [28]. However, the function of TNFRSF12A in MI progression has yet to be elucidated. We established a mouse MI model by LAD ligation. MI mice exhibited significantly impaired systolic function (reduced LVEF/LVFS) and ventricular dilation (elevated LVIDs, LVIDd, LVESV, and LVEDV) versus sham controls (Figure 9a,b). Meanwhile, we observed that upregulation of TNFRSF12A in protein and mRNA levels in the MI group (Figure 9c,d). We used TGF*β* to induce activation of NIH3T3, which showed that TNFRSF12A was upregulated under TGF*β* stimulation (Figure 9e). These results indicated that TNFRSF12A might modulate fibrosis in fibroblasts.

Figure 9Experiment validation. (a) Representative B‐mode (left) and M‐mode (right) images across experimental groups at 28d post‐MI. (b) Quantification of functional parameters (LVEF, LVFS) and structural remodeling indices (LVEDV, LVESV, LVIDd, and LVIDs). (c) Western blots and quantitative analysis of TNFRSF12A expression. (d) mRNA level of Tnfrsf12a. (e) Western blots and quantitative analysis of TNFRSF12A expression in NIH3T3 without and with TGF*β*.

**Figure:**
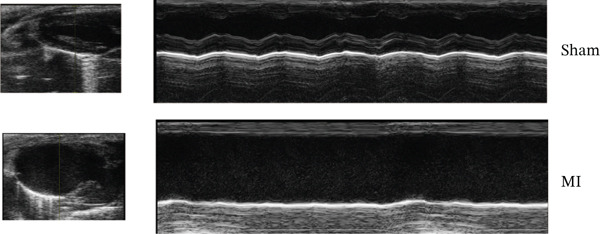
(a)

**Figure:**
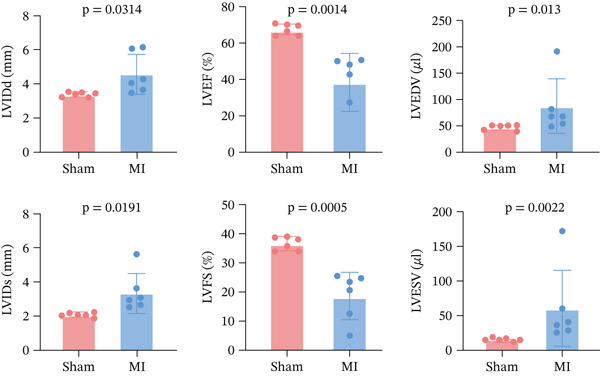
(b)

**Figure:**
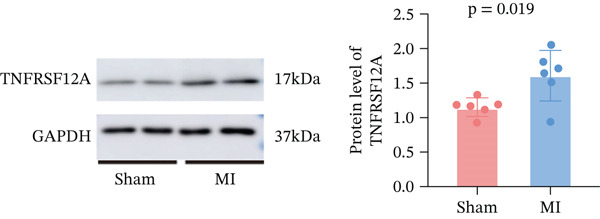
(c)

**Figure:**
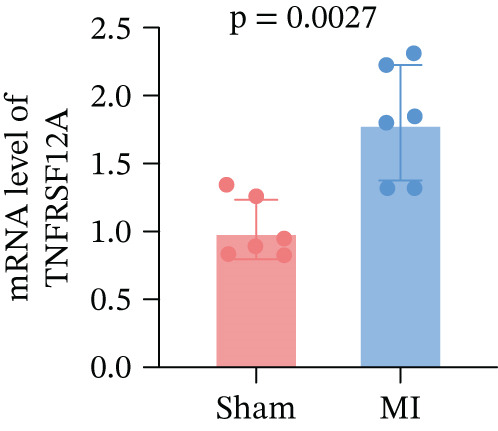
(d)

**Figure:**
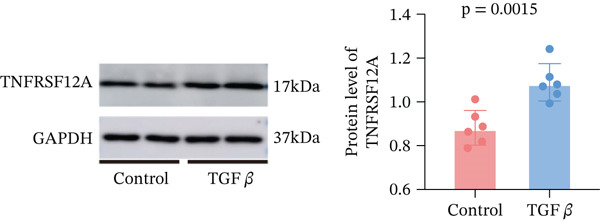
(e)

## 4. Discussion

The increasing prevalence of MI has imposed a significant burden on global healthcare systems [24]. Despite significant advances in understanding MI, current clinical treatment methods remain limited. Therefore, novel therapeutic approaches are urgently needed.

MI, triggered by coronary artery obstruction, lead to ischemic myocardial injury and cardiomyocyte apoptosis, deteriorating heart failure [29]. However, noncardiomyocytes, such as fibroblasts, endothelial cells, adipocytes, and immune cells account for approximately of the total cells, contributing to maintain cardiac structure, function, and repair. Emerging evidence highlights noncardiomyocyte apoptosis as a key driver of MI [30].

Noncardiomyocytes apoptosis alters the cellular composition, microenvironment, cardiomyocyte survival and cardiac repair through regulating processes such as inflammatory responses, extracellular matrix remodeling, angiogenesis, and fibrosis [5]. For instance, macrophage depletion facilitated impaired clearance of cellular debris, leading to continuous inflammation, which compromised macrophage repair effect and exacerbated the progression of heart failure [10]. Fibroblast apoptosis disrupted secretion of extracellular matrix, fibrosis formation, and integrity of the heart [31]. Following MI, apoptotic endothelial cells release soluble pro‐apoptotic mediators, inducing cardiomyocyte apoptosis. Therefore, preventing endothelial cell apoptosis could protect cardiomyocyte [11]. Notably, noncardiomyocyte apoptosis activity may far exceed that of cardiomyocytes, underscoring its potential therapeutic role in the pathophysiology of MI.

Despite previous studies highlighting the role of noncardiomyocytes in MI, limited technological methods still hinder understanding of core mechanisms. Hence, the detailed regulating function of noncardiomyocytes remains obscure. By integrating high throughput sequencing (snRNA‐seq and bulk seq) and bioinformatics analysis, we revealed the potential role of noncardiocyte apoptosis. These results demonstrate that noncardiocyte apoptosis might be a potential target for MI.

Single‐cell seq analysis enables researchers to identify key molecular at single cell level resolution. Here, we integrated snRNA‐seq and bulk RNA‐seq data through bioinformatics analysis, identifying TNFRSF12A as a key regulator among ARGs in MI. Our findings suggest that TNFRSF12A^+^ fibroblasts might represent a promising therapeutic target for mitigating MI‐related damage. To validate these findings, we confirmed the upregulation of TNFRSF12A at both the protein and mRNA levels in a murine MI model and in TGF*β* induced fibroblasts. Fibroblasts serve as the primary effector cells in cardiac fibrosis, and their activation is directly linked to adverse remodeling post‐MI, whereas the pathological role of adipocytes in the myocardium remains debated [32–35]. Future studies could explore the potential role of TNFRSF12A in adipocytes, particularly its association with epicardial adipose tissue (EAT) inflammation, which may require the development of more specific adipocyte models.

TNFRSF12A, a TNF receptor superfamily member, is implicated in cardiac fibrosis, and its circulating levels are positively correlated with the severity of heart failure [36]. Additionally, recombinant TNFRSF12A protein can activate cytokine synthesis and collagen expression in cardiac fibroblasts through the NF‐*κ*B pathway. However, studies on the role of TNFRSF12A in cardiovascular diseases remain limited. During cardiac remodeling, TNFRSF12A is significantly upregulated, yet its specific functional contributions remain unclear.

Our study employed three distinct machine‐learning algorithms (RF, Boruta, and LASSO) to identify overlapping hub ARGs. This strategy might be too conservative to exclude other potential genes identified by two algorithms. However, our primary objective was to pinpoint the most prioritized genes. Meanwhile, we recognize that genes identified by two algorithms represent potential candidates to evaluate their potential biological significance in future investigation.

Through integrative analysis of snRNA‐seq and bulk RNA‐seq datasets augmented by machine learning approaches, we identified TNFRSF12A as a novel diagnostic biomarker for MI. Functional characterization revealed that TNFRSF12A^+^ fibroblasts display distinct molecular signatures, with significant enrichment in: NOD‐like receptor signaling pathways (KEGG), toll‐like receptor cascades (Reactome), mitochondrial electron transport (Reactome). While our experimental validation confirmed the upregulation of TNFRSF12A in MI and in TGF*β* induced fibroblasts, the precise molecular mechanisms by which TNFRSF12A modulates apoptosis in fibroblasts remain unclear. Although previous studies have used NIH3T3 cells for in vitro experiments on cardiac fibrosis, we acknowledge that embryonic NIH3T3 cells may differ from adult cardiac myofibroblasts in: maturation state and epigenetic programming, stress response pathways, and extracellular matrix production profiles [37, 38]. These differences could affect the translational relevance of our in vitro findings. We will consider using primary adult cardiac fibroblasts for future, more translationally relevant validation experiments. We will further explore TNFRSF12A′s regulatory role in fibroblasts during MI. Additionally, clinical observation is essential to assess the translational potential of TNFRSF12A as a therapeutic target.

In conclusion, our findings revealed TNFRSF12A as a key regulator in MI, with potential implications for diagnosis and treatment.

However, given the complex molecular mechanisms post MI, further in‐depth studies are required to elucidate the biological significance of TNFRSF12A′s role in fibroblasts during MI. Above process might identify new therapeutic approach to benefit MI patients.

## Author Contributions

**Bin Li:** project administration, data, formal analysis. **Yuan Wu:** software, supervision, validation. **Shude Liao:** investigation, methodology. **Jing Wang:** analysis, funding, writing. **Chenghui Yan:** resources, software. **Panting Wei:** software, supervision. **Dali Zhang:** resources, software. **Dan Liu**: supervision, validation. **Yaling Han:** analysis, funding, investigation.

## Funding

This study was supported by National Natural Science Foundation of China (82470303, 82570354, 82270300).

## Disclosure

The authors take full responsibility for the content of the publication.

## Ethics Statement

We confirmed that all the experiments in this study were performed in accordance with the relevant guidelines and regulations. All the procedures of the study followed the ARRIVE guidelines.

## Consent

The authors have nothing to report.

## Conflicts of Interest

The authors declare no conflicts of interest.

## Supporting Information

Additional supporting information can be found online in the Supporting Information section.

## Supporting information


**Supporting Information 1** Supporting information.pdf lists that the full unedited membrane represents the representative pictures used in Figures 9c and 9e of the manuscript.

**Supporting Information 2** Table S1.xlsx lists detailed statistical analysis.

## Data Availability

The transcriptomic datasets in this study are publicly accessible through the Gene Expression Omnibus (GEO) under accession codes GSE21610 and GSE270788.
