# Supplementary figures and images for: Machine Learning Integrates Bulk and Single‐Nucleus RNA Sequence to Explore Apoptosis‐Related Gene in Myocardial Infarction
Source: Cardiovasc Ther. 2026 Mar 18;2026:5553167. doi: 10.1155/cdr/5553167 (PMC13140870; doi:10.1155/cdr/5553167)

Fig.9C

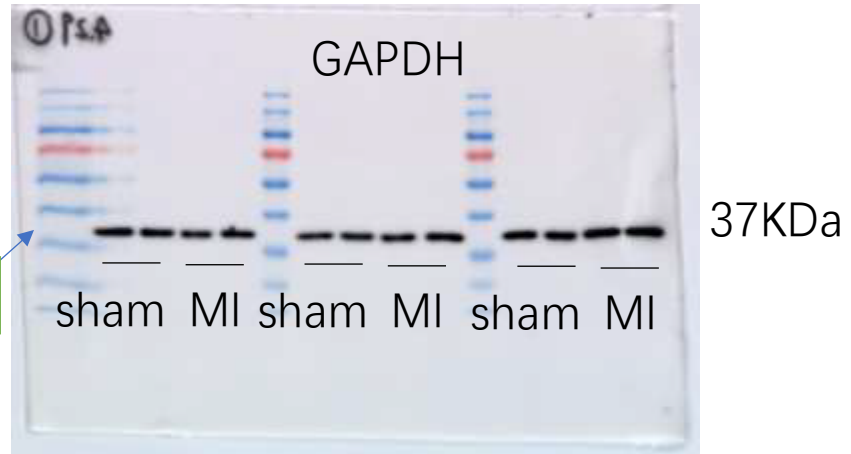

stripping

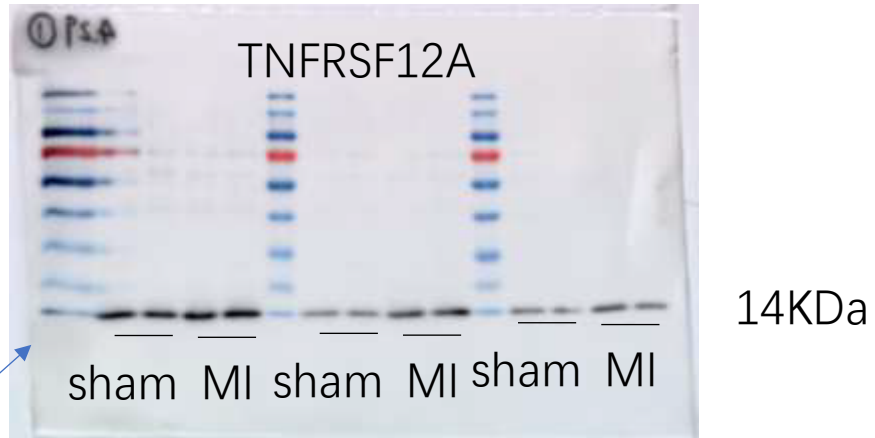

Fig.9E

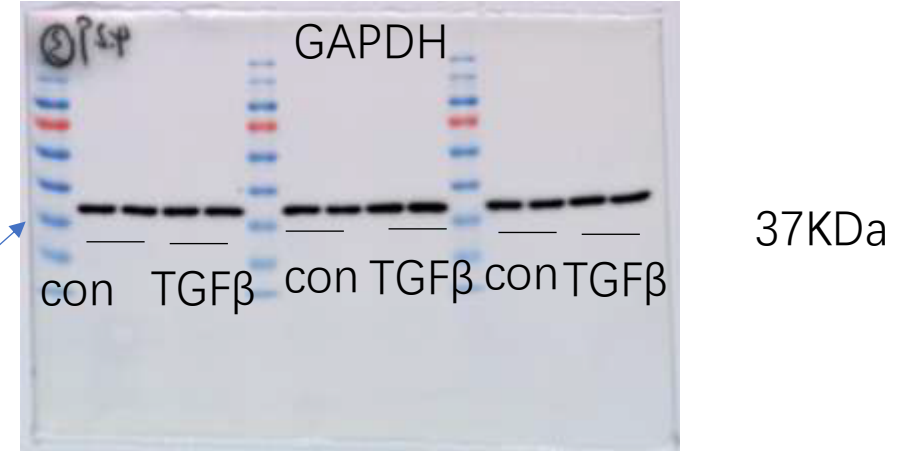

stripping

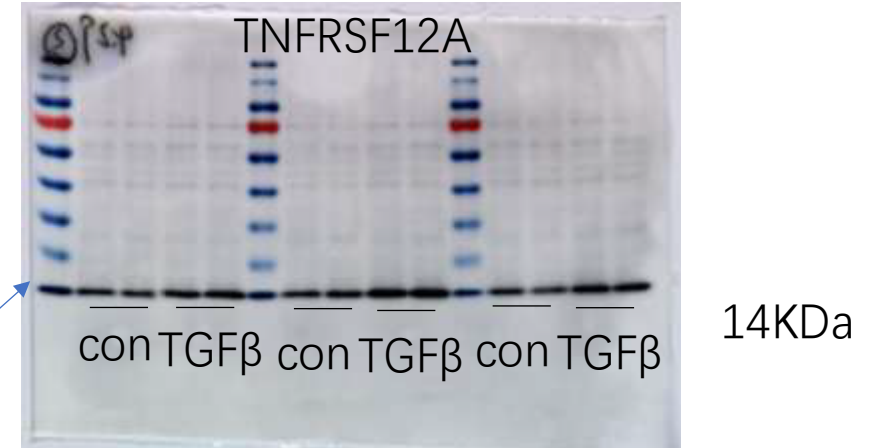

Supplement: Supplementary file 1 — Supporting Information 1 Supporting information.pdf lists that the full unedited membrane represents the representative pictures used in Figures 9c and 9e of the manuscript. [file CDR-2026-5553167-s002.pdf]
