# Supplementary material for: Machine Learning Integrates Bulk and Single‐Nucleus RNA Sequence to Explore Apoptosis‐Related Gene in Myocardial Infarction
Source: Cardiovasc Ther. 2026 Mar 18;2026:5553167. doi: 10.1155/cdr/5553167 (PMC13140870; doi:10.1155/cdr/5553167)
Supplement: Supplementary file 2 — Supporting Information 2 Table S1.xlsx lists detailed statistical analysis. [file CDR-2026-5553167-s001.pdf]

Figure	comparison	Statistical test	P-value	data	
Fig.4B	Control vs MI	Wilcoxon Test	< 2.2e^-16	Control 0.1104(0.09369-0.1293)	MI 0.1286(0.1083-0.1571)
Fig.4C	Control Adipocytes vs MI Adipocytes	Wilcoxon Test	< 2e^-16	Control Adipocytes 0.12(0.09-0.15)	MI Adipocytes 0.17(0.11-0.23)
	Control Cardiomyocytes vs MI Cardiomyocytes	Wilcoxon Test	1e^-09	Control Cardiomyocytes 0.11(0.07-0.15)	MI Cardiomyocytes 0.11(0.07-0.15)
	Control Endothelium vs MI Endothelium	Wilcoxon Test	< 2e^-16	Control Endothelium 0.11(0.08-0.14)	MI Endothelium 0.12(0.08-0.16)
	Control Fibroblasts vs MI Fibroblasts	Wilcoxon Test	< 2e^-16	Control Fibroblasts 0.12(0.08-0.16)	MI Fibroblasts 0.14(0.09-0.19)
	Control Myeloid vs MI Myeloid	Wilcoxon Test	3.1e^-11	Control Myeloid 0.1(0.07-0.13)	MI Myeloid 0.12(0.09-0.15)
	Control SMC vs MI SMC	Wilcoxon Test	0.089	Control SMC 0.11(0.07-0.15)	MI SMC 0.12(0.09-0.15)
	MI Fibroblasts vs MI Cardiomyocytes	Wilcoxon Test	< 2.2e^-16	MI Fibroblasts 1.07(0.67-1.47)	MI Cardiomyocytes 0.84(0.55-1.13)
	MI Fibroblasts vs MI endotheliums	Wilcoxon Test	< 2.2e^-16	MI Fibroblasts 1.07(0.67-1.47)	MI Endotheliums 0.93(0.66-0.13)
	MI Fibroblasts vs MI Adipocytes	Wilcoxon Test	< 2.2e^-16	MI Fibroblasts 1.07(0.67-1.47)	MI Adipocytes 1.3(0.86-1.74)
	MI Fibroblasts vs MI Cardomyocytes	Wilcoxon Test	< 2.2e^-16	MI Fibroblasts 1.07(0.67-1.47)	MI Myeloid 0.86 (0.66-1.06)
	MI Fibroblasts vs MI Cardomyocytes	Wilcoxon Test	< 2.2e^-16	MI Fibroblasts 1.07(0.67-1.47)	MI SMC 0.87(0.64-1.1)
Fig.7C	TNFRSF12A expression in Control vs MI	Wilcoxon Test	0.0009	8.0(7.7-8.4)	8.9(8.16-9.64)
Fig.9B	LVEF	Unpaired two-tailed Student's t-test	0.0014	67.48±3.108	38.34±16.05
	LVFS	Unpaired two-tailed Student's t-test	0.0005	36.61 ± 2.391	18.71 ± 8.261
	LVIDd	Unpaired two-tailed Student's t-test	0.0314	3.405 ± 0.1408	4.632 ± 1.193
	LVIDs	Unpaired two-tailed Student's t-test	0.0191	2.17 ± 0.1273	3.552 ± 1.206
	LVEDV	Mann-Whitney test	0.013	48.31 ± 4.977	88.01 ± 52.42
	LVESV	Mann-Whitney test	0.0022	15.72 ± 2.259	60.63 ± 55.71
Fig.9D	mRNA level of TNFRSF12A in sham and MI mouse	Unpaired two-tailed Student's t-test	0.0027	1.02 ± 0.2284	1.802 ± 0.4272
Fig.9C	protein level of TNFRSF12A in sham and MI mouse	Unpaired two-tailed Student's t-test	0.019	1.154 ± 0.1304	1.61 ± 0.3779
Fig.9E	protein level of TNFRSF12A in Control and TGF-β group	Unpaired two-tailed Student's t-test	0.0015	0.8836 ± 0.08129	1.091 ± 0.08482

Figure	sample	Statistical test	Correlation Coefficient	P-value
Fig.7B	Activated B cell	spearman	-0.17	0.59
	Activated CD4 T cell	spearman	0.38	0.22
	Activated CD8 T cell	spearman	-0.33	0.30
	Activated dendritic cell	spearman	-0.34	0.29
	CD56bright natural killer cell	spearman	-0.30	0.34
	CD56dim natural killer cell	spearman	-0.13	0.70
	Central memory CD4 T cell	spearman	-0.23	0.47
	Central memory CD8 T cell	spearman	-0.41	0.19
	Effector memeory CD4 T cell	spearman	0.60	0.04
	Effector memeory CD8 T cell	spearman	0.52	0.08
	Eosinophil	spearman	0.24	0.46
	Gamma delta T cell	spearman	-0.14	0.66
	Immature B cell	spearman	0.01	0.97
	Immature dendritic cell	spearman	0.73	0.01
	Macrophage	spearman	-0.31	0.33
	Mast cell	spearman	-0.11	0.73
	MDSC	spearman	-0.13	0.68
	Memory B cell	spearman	0.20	0.54
	Monocyte	spearman	0.14	0.66
	Natural killer cell	spearman	0.50	0.10
	Natural killer T cell	spearman	0.27	0.39
	Neutrophil	spearman	0.29	0.37
	Plasmacytoid dendritic cell	spearman	0.10	0.76
	Regulatory T cell	spearman	-0.55	0.06
	T follicular helper cell	spearman	-0.33	0.30
	Type 1 T helper cell	spearman	0.34	0.28
	Type 17 T helper cell	spearman	0.01	0.97
	Type 2 T helper cell	spearman	0.09	0.78
